# (*E*)-3,4,5-Trimeth­oxy-*N*′-[(6-meth­oxy-4-oxo-4*H*-chromen-3-yl)methyl­idene]benzohydrazide monohydrate

**DOI:** 10.1107/S1600536814014937

**Published:** 2014-07-02

**Authors:** Yoshinobu Ishikawa, Kohzoh Watanabe

**Affiliations:** a School of Pharmaceutical Sciences, University of Shizuoka, 52-1 Yada, Suruga-ku, Shizuoka 422-8526, Japan

**Keywords:** crystal structure

## Abstract

In the title chromone-tethered benzohydrazide derivative, C_21_H_20_N_2_O_7_·H_2_O, the atoms of the 4*H*-chromen-4-one segment are essentially coplanar (r.m.s. deviation = 0.0073 Å) with the largest deviation from the mean plane [0.012 (3) Å] being found for the benzene C atom. The dihedral angles between the chromone segment and the hydrazide plane and between the chromone segment and the benzene ring of the tri­meth­oxy­benzene unit are 24.67 (9) and 41.28 (8) Å, respectively. The mol­ecule is connected to the solvent water mol­ecule by O—H⋯O hydrogen bonds and weak C—H⋯O inter­actions. Additional N—H⋯O inter­actions are observed and together they link the mol­ecules into chains forming a two-dimensional network along (011).

## Related literature   

For the biological activity of related compounds, see: Khan *et al.* (2009 ▶); Tu *et al.* (2013 ▶). For related structures, see: Ishikawa *et al.* (2014*a* ▶,*b* ▶).
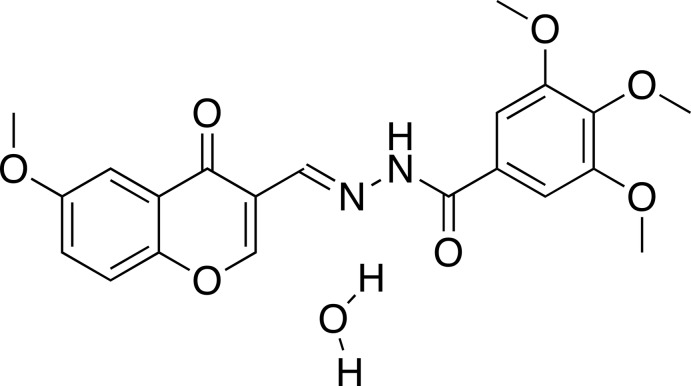


## Experimental   

### 

#### Crystal data  

C_21_H_20_N_2_O_7_·H_2_O
*M* *_r_* = 430.41
Triclinic, 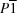

*a* = 7.782 (3) Å
*b* = 9.015 (5) Å
*c* = 14.991 (6) Å
α = 103.17 (5)°
β = 96.51 (3)°
γ = 95.52 (4)°
*V* = 1009.4 (8) Å^3^
*Z* = 2
Mo *K*α radiation
μ = 0.11 mm^−1^
*T* = 100 K
0.30 × 0.20 × 0.18 mm

#### Data collection  

Rigaku AFC-7R diffractometer
5621 measured reflections
4621 independent reflections
3518 reflections with *F* ^2^ > 2σ(*F* ^2^)
*R*_int_ = 0.111
3 standard reflections every 150 reflections intensity decay: 2.3%

#### Refinement  

*R*[*F* ^2^ > 2σ(*F* ^2^)] = 0.076
*wR*(*F* ^2^) = 0.230
*S* = 1.02
4621 reflections
292 parameters
H-atom parameters constrained
Δρ_max_ = 0.65 e Å^−3^
Δρ_min_ = −0.57 e Å^−3^

### 

Data collection: *WinAFC Diffractometer Control Software* (Rigaku, 1999 ▶); cell refinement: *WinAFC Diffractometer Control Software*; data reduction: *WinAFC Diffractometer Control Software*; program(s) used to solve structure: *SIR2008* (Burla *et al.*, 2007 ▶); program(s) used to refine structure: *SHELXL97* (Sheldrick, 2008 ▶); molecular graphics: *CrystalStructure* (Rigaku, 2010 ▶); software used to prepare material for publication: *CrystalStructure*.

## Supplementary Material

Crystal structure: contains datablock(s) General, I. DOI: 10.1107/S1600536814014937/jj2189sup1.cif

Structure factors: contains datablock(s) I. DOI: 10.1107/S1600536814014937/jj2189Isup2.hkl

Supporting information file. DOI: 10.1107/S1600536814014937/jj2189Isup3.cml

CCDC reference: 1010096

Additional supporting information:  crystallographic information; 3D view; checkCIF report

## Figures and Tables

**Table 1:** Hydrogen-bond geometry (Å, °)

*D*—H⋯*A*	*D*—H	H⋯*A*	*D*⋯*A*	*D*—H⋯*A*
O8—H21⋯O2^i^	0.91	1.93	2.829 (4)	168
O8—H22⋯O4	0.83	2.27	3.036 (3)	153
N2—H9⋯O6^ii^	0.88	2.28	3.062 (3)	149
C1—H1⋯O8	0.95	2.30	3.214 (4)	161
C4^i^—H2^i^⋯O8	0.95	2.60	3.489 (4)	155

Symmetry codes: (i) 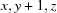; (ii) 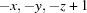.

**Fig. 1.**
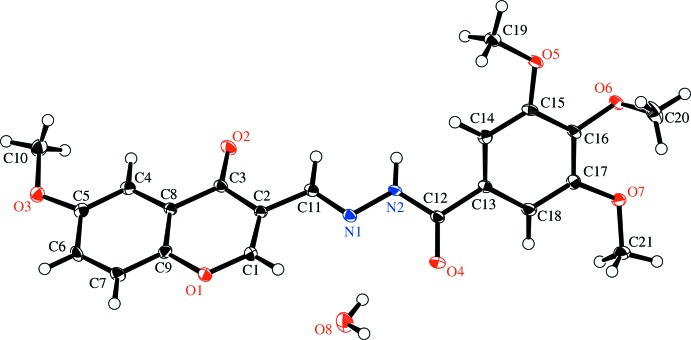
Fig. .1 The molecular structure of the title compound, with displacement ellipsoids drawn at the 50% probability level. Hydrogen atoms are shown as small spheres of arbitrary radius.

**Fig. 2.**
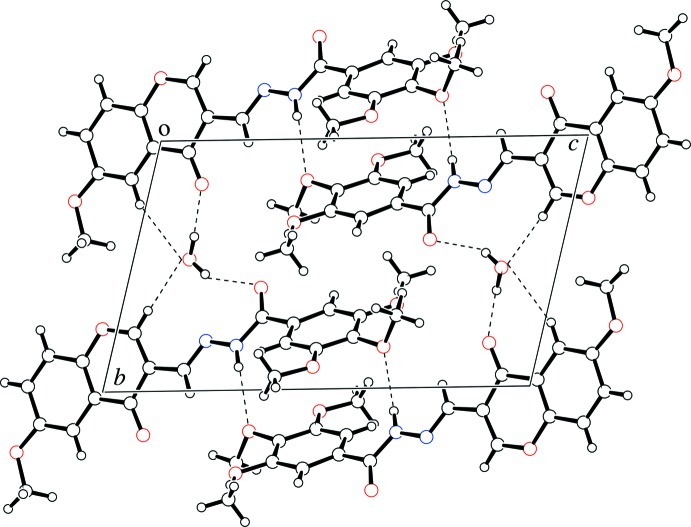
Crystal packing of the title compound viewed along the a axis. O–H···O hydrogen bonds and weak O—H···O, N—H···O, C–H···O intermolecular interactions are represented by dashed lines.

## supplementary crystallographic information

### S1. Comment

Schiff base derivatives of 3-formyl chromones have attracted much attention due to their biological functions such as enzyme inhibition (Khan *et al.* 2009; Tu *et al.* 2013). We herein report the crystal structure of the title compound, which was obtained from the condensation reaction of 6-methoxy-3-formylchromone with 3,4,5-trimethoxybenzoylhydrazide in benzene.

The mean deviation of the least-square planes for the non-hydrogen atoms of the 4*H*-chromen-4-one segment is 0.0073 Å, and the largest deviation is 0.012 (3) Å for C6 (Fig.1) showing an essentially coplanar segment. The dihedral angles between this chromone segment and the hydrazide (N1/N2/C12/O4) plane and between the chromone segment and the benzene ring of the trimethoxybenzene unit are 24.67 (9) Å and 41.28 (8) Å, respectively. In the crystal, the molecule is connected to the solvent water molecule by O–H···O hydrogen bonds and weak O—H···O, C–H···O intermolecular interactions (Table 1). Additional weak N—H···O intermolecular interactions are observed and together they link the molecules into chains forming a 2-D network along (011) (Fig. 2).

### S2. Experimental

3,4,5-Trimethoxybenzoylhydrazide (1.00 mmol), 6-methoxy-3-formylchromone (1.00mmol), and a few drops of acetic acid were dissolved in 25 ml of benzene, andthe mixture was refluxed with Dean-Stark apparatus for 6 h. After cooling, theprecipitates were collected, washed with *n*-hexane, and dried (yield 63.6%). Single crystals suitable for X-ray diffraction were obtained by slow evaporation of an acetonitrile solution of the title compound at room temperature. DART-MS calcd for [C_21_H_20_N_2_O_7_ + H^+^]: 413.135, found 413.158.

### S3. Refinement

The C(*sp*^2^)- and N(*sp*^2^)-bound hydrogen atoms were placed in geometrical positions [C–H 0.95 Å, *U*_iso_(H) = 1.2*U*_eq_(C), N–H 0.88 Å, *U*_iso_(H) = 1.2*U*_eq_(N)], and refined using a riding model. Hydrogen atoms of methyl groups were found in a difference Fourier map, and a rotating group model was applied with distance constraint [C–H = 0.98 Å, *U*_iso_(H) = 1.2*U*_eq_(C)]. Hydrogen atoms of the water molecule were found in a difference Fourier map, and were refined using a riding model.

### Crystal data

**Table:**

C_21_H_20_N_2_O_7_·H_2_O	*Z* = 2
*M**_r_* = 430.41	*F*(000) = 452.00
Triclinic, *P*1	*D*_x_ = 1.416 Mg m^−^^3^
Hall symbol: -P 1	Mo *K*α radiation, λ = 0.71069 Å
*a* = 7.782 (3) Å	Cell parameters from 25 reflections
*b* = 9.015 (5) Å	θ = 15.9–17.4°
*c* = 14.991 (6) Å	µ = 0.11 mm^−^^1^
α = 103.17 (5)°	*T* = 100 K
β = 96.51 (3)°	Block, colorless
γ = 95.52 (4)°	0.30 × 0.20 × 0.18 mm
*V* = 1009.4 (8) Å^3^	

### Data collection

**Table:**

Rigaku AFC-7R diffractometer	θ_max_ = 27.5°
ω–2θ scans	*h* = −10→10
5621 measured reflections	*k* = −11→6
4621 independent reflections	*l* = −18→19
3518 reflections with *F*^2^ > 2σ(*F*^2^)	3 standard reflections every 150 reflections
*R*_int_ = 0.111	intensity decay: 2.3%

### Refinement

**Table:**

Refinement on *F*^2^	Secondary atom site location: difference Fourier map
*R*[*F*^2^ > 2σ(*F*^2^)] = 0.076	Hydrogen site location: inferred from neighbouring sites
*wR*(*F*^2^) = 0.230	H-atom parameters constrained
*S* = 1.02	*w* = 1/[σ^2^(*F*_o_^2^) + (0.1645*P*)^2^ + 0.3451*P*] where *P* = (*F*_o_^2^ + 2*F*_c_^2^)/3
4621 reflections	(Δ/σ)_max_ < 0.001
292 parameters	Δρ_max_ = 0.65 e Å^−^^3^
0 restraints	Δρ_min_ = −0.57 e Å^−^^3^
Primary atom site location: structure-invariant direct methods	

### Special details

**Table:**

Experimental. ^1^H NMR (400 MHz, DMSO-*d*_6_): *δ* = 3.73 (s, 3H), 3.88 (s, 6H), 3.89 (s, 3H), 7.27 (s, 2H), 7.46 (dd, 1H, *J* = 2.9 and 9.3 Hz), 7.51 (d, 1H, *J* = 2.9 Hz), 7.72 (d, 1H, *J* = 9.3 Hz), 8.68 (s, 1H), 8.85 (s, 1H), 11.80 (s, 1H).
Refinement. Refinement was performed using all reflections. The weighted *R*-factor (*wR*) and goodness of fit (*S*) are based on *F*^2^. *R*-factor (gt) are based on *F*. The threshold expression of *F*^2^ > 2.0 σ(*F*^2^) is used only for calculating *R*-factor (gt).

### Fractional atomic coordinates and isotropic or equivalent isotropic displacement parameters (Å^2^)

**Table:**

	*x*	*y*	*z*	*U*_iso_*/*U*_eq_	
O1	0.7962 (3)	0.25163 (18)	1.03469 (11)	0.0170 (4)	
O2	0.5804 (3)	−0.17435 (19)	0.88102 (12)	0.0192 (4)	
O3	0.9883 (3)	−0.23616 (19)	1.16901 (11)	0.0182 (4)	
O4	0.4338 (3)	0.4081 (2)	0.68493 (12)	0.0217 (4)	
O5	−0.2787 (2)	0.0891 (2)	0.52041 (11)	0.0183 (4)	
O6	−0.2145 (2)	0.16565 (18)	0.36535 (11)	0.0168 (4)	
O7	0.0852 (3)	0.3332 (2)	0.35685 (11)	0.0206 (4)	
O8	0.6699 (3)	0.5255 (3)	0.86847 (14)	0.0302 (5)	
N1	0.4696 (3)	0.1985 (3)	0.78507 (12)	0.0140 (4)	
N2	0.3390 (3)	0.1774 (2)	0.71139 (13)	0.0135 (4)	
C1	0.6843 (3)	0.2331 (3)	0.95678 (15)	0.0154 (5)	
C2	0.6093 (3)	0.0946 (3)	0.90324 (14)	0.0127 (5)	
C3	0.6469 (3)	−0.0464 (3)	0.92841 (14)	0.0129 (5)	
C4	0.8169 (3)	−0.1504 (3)	1.04813 (15)	0.0140 (5)	
C5	0.9310 (3)	−0.1250 (3)	1.12933 (15)	0.0143 (5)	
C6	0.9961 (3)	0.0268 (3)	1.17845 (15)	0.0161 (5)	
C7	0.9504 (3)	0.1495 (3)	1.14648 (15)	0.0168 (5)	
C8	0.7690 (3)	−0.0245 (3)	1.01460 (14)	0.0121 (5)	
C9	0.8361 (3)	0.1234 (3)	1.06352 (15)	0.0135 (5)	
C10	0.9139 (4)	−0.3915 (3)	1.12624 (18)	0.0228 (6)	
C11	0.4829 (3)	0.0839 (3)	0.82108 (14)	0.0130 (5)	
C12	0.3287 (3)	0.2913 (3)	0.66582 (15)	0.0133 (5)	
C13	0.1788 (3)	0.2631 (3)	0.58907 (15)	0.0130 (5)	
C14	0.0185 (3)	0.1864 (3)	0.59606 (15)	0.0140 (5)	
C15	−0.1154 (3)	0.1596 (3)	0.52197 (15)	0.0136 (5)	
C16	−0.0862 (3)	0.2063 (3)	0.44125 (15)	0.0142 (5)	
C17	0.0733 (3)	0.2886 (3)	0.43727 (15)	0.0157 (5)	
C18	0.2067 (3)	0.3176 (3)	0.51160 (15)	0.0155 (5)	
C19	−0.3186 (3)	0.0514 (3)	0.60432 (16)	0.0193 (5)	
C20	−0.3090 (4)	0.2905 (4)	0.35235 (19)	0.0297 (7)	
C21	0.2353 (4)	0.4363 (4)	0.35433 (17)	0.0250 (6)	
H1	0.6559	0.3225	0.9380	0.0184*	
H2	0.7713	−0.2522	1.0152	0.0168*	
H3	1.0731	0.0437	1.2347	0.0193*	
H4	0.9952	0.2512	1.1799	0.0202*	
H5A	0.9310	−0.4163	1.0610	0.0273*	
H6B	0.7888	−0.4030	1.1307	0.0273*	
H7C	0.9709	−0.4614	1.1578	0.0273*	
H8	0.4097	−0.0099	0.7941	0.0156*	
H9	0.2647	0.0928	0.6944	0.0162*	
H10	0.0007	0.1530	0.6504	0.0168*	
H11	0.3152	0.3739	0.5094	0.0186*	
H12A	−0.2449	−0.0245	0.6190	0.0231*	
H13B	−0.4415	0.0086	0.5963	0.0231*	
H14C	−0.2966	0.1443	0.6550	0.0231*	
H15A	−0.3884	0.2583	0.2939	0.0357*	
H16B	−0.2266	0.3788	0.3506	0.0357*	
H17C	−0.3763	0.3193	0.4037	0.0357*	
H18A	0.3401	0.3852	0.3603	0.0300*	
H19B	0.2448	0.5277	0.4055	0.0300*	
H20C	0.2238	0.4668	0.2954	0.0300*	
H21	0.6256	0.6158	0.8696	0.0244*	
H22	0.6069	0.4653	0.8237	0.0513*	

### Atomic displacement parameters (Å^2^)

**Table:**

	*U*^11^	*U*^22^	*U*^33^	*U*^12^	*U*^13^	*U*^23^
O1	0.0212 (9)	0.0130 (8)	0.0137 (8)	−0.0027 (6)	−0.0035 (7)	0.0019 (6)
O2	0.0221 (9)	0.0137 (8)	0.0171 (8)	−0.0023 (7)	−0.0051 (7)	−0.0001 (6)
O3	0.0233 (9)	0.0160 (9)	0.0139 (8)	0.0005 (7)	−0.0003 (7)	0.0031 (6)
O4	0.0215 (9)	0.0183 (9)	0.0213 (9)	−0.0093 (7)	−0.0059 (7)	0.0060 (7)
O5	0.0122 (8)	0.0263 (9)	0.0138 (8)	−0.0063 (7)	−0.0002 (6)	0.0041 (7)
O6	0.0193 (9)	0.0155 (8)	0.0109 (8)	−0.0032 (7)	−0.0034 (6)	−0.0015 (6)
O7	0.0243 (9)	0.0239 (9)	0.0103 (8)	−0.0108 (7)	−0.0019 (7)	0.0049 (7)
O8	0.0449 (12)	0.0144 (9)	0.0239 (10)	−0.0013 (9)	−0.0122 (9)	0.0000 (8)
N1	0.0133 (9)	0.0170 (9)	0.0098 (9)	−0.0019 (7)	0.0009 (7)	0.0012 (7)
N2	0.0135 (9)	0.0127 (9)	0.0113 (9)	−0.0045 (7)	−0.0014 (7)	0.0013 (7)
C1	0.0170 (11)	0.0151 (11)	0.0130 (10)	−0.0007 (9)	−0.0010 (8)	0.0040 (8)
C2	0.0126 (10)	0.0150 (11)	0.0095 (10)	−0.0020 (8)	0.0013 (8)	0.0023 (8)
C3	0.0127 (10)	0.0141 (11)	0.0103 (10)	−0.0010 (8)	0.0014 (8)	0.0007 (8)
C4	0.0152 (11)	0.0140 (11)	0.0114 (10)	−0.0006 (8)	0.0034 (8)	0.0003 (8)
C5	0.0146 (11)	0.0177 (11)	0.0120 (10)	0.0026 (8)	0.0060 (8)	0.0040 (8)
C6	0.0165 (11)	0.0207 (12)	0.0086 (10)	−0.0011 (9)	−0.0022 (8)	0.0016 (8)
C7	0.0204 (12)	0.0153 (11)	0.0105 (10)	−0.0034 (9)	−0.0007 (9)	−0.0020 (8)
C8	0.0116 (10)	0.0149 (11)	0.0086 (10)	−0.0008 (8)	0.0019 (8)	0.0014 (8)
C9	0.0164 (11)	0.0123 (10)	0.0117 (10)	0.0001 (8)	0.0032 (8)	0.0031 (8)
C10	0.0306 (14)	0.0144 (12)	0.0222 (12)	0.0013 (10)	−0.0013 (10)	0.0051 (9)
C11	0.0129 (10)	0.0150 (11)	0.0094 (10)	−0.0009 (8)	0.0024 (8)	−0.0001 (8)
C12	0.0128 (10)	0.0138 (10)	0.0112 (10)	−0.0014 (8)	0.0006 (8)	0.0008 (8)
C13	0.0159 (11)	0.0095 (10)	0.0107 (10)	−0.0008 (8)	−0.0007 (8)	−0.0013 (8)
C14	0.0162 (11)	0.0135 (10)	0.0112 (10)	0.0008 (8)	0.0031 (8)	0.0007 (8)
C15	0.0131 (10)	0.0113 (10)	0.0136 (10)	−0.0019 (8)	0.0023 (8)	−0.0018 (8)
C16	0.0149 (11)	0.0121 (10)	0.0115 (10)	−0.0005 (8)	−0.0023 (8)	−0.0024 (8)
C17	0.0213 (12)	0.0145 (10)	0.0091 (10)	−0.0009 (9)	0.0014 (9)	0.0002 (8)
C18	0.0161 (11)	0.0150 (11)	0.0127 (10)	−0.0035 (8)	0.0000 (9)	0.0008 (8)
C19	0.0142 (11)	0.0276 (13)	0.0155 (11)	−0.0035 (9)	0.0049 (9)	0.0051 (9)
C20	0.0321 (15)	0.0259 (14)	0.0234 (13)	0.0072 (11)	−0.0108 (11)	−0.0044 (11)
C21	0.0240 (13)	0.0315 (14)	0.0171 (12)	−0.0119 (11)	−0.0010 (10)	0.0096 (10)

### Geometric parameters (Å, º)

**Table:**

O1—C1	1.343 (3)	C13—C18	1.391 (4)
O1—C9	1.373 (3)	C14—C15	1.394 (3)
O2—C3	1.238 (3)	C15—C16	1.403 (4)
O3—C5	1.362 (4)	C16—C17	1.398 (4)
O3—C10	1.435 (3)	C17—C18	1.393 (3)
O4—C12	1.226 (3)	O8—H21	0.911
O5—C15	1.360 (3)	O8—H22	0.832
O5—C19	1.434 (4)	N2—H9	0.880
O6—C16	1.381 (3)	C1—H1	0.950
O6—C20	1.440 (4)	C4—H2	0.950
O7—C17	1.364 (4)	C6—H3	0.950
O7—C21	1.431 (4)	C7—H4	0.950
N1—N2	1.379 (3)	C10—H5A	0.980
N1—C11	1.277 (4)	C10—H6B	0.980
N2—C12	1.361 (4)	C10—H7C	0.980
C1—C2	1.357 (3)	C11—H8	0.950
C2—C3	1.451 (4)	C14—H10	0.950
C2—C11	1.465 (3)	C18—H11	0.950
C3—C8	1.477 (3)	C19—H12A	0.980
C4—C5	1.382 (3)	C19—H13B	0.980
C4—C8	1.407 (4)	C19—H14C	0.980
C5—C6	1.412 (4)	C20—H15A	0.980
C6—C7	1.363 (4)	C20—H16B	0.980
C7—C9	1.403 (3)	C20—H17C	0.980
C8—C9	1.387 (3)	C21—H18A	0.980
C12—C13	1.502 (3)	C21—H19B	0.980
C13—C14	1.394 (4)	C21—H20C	0.980
			
O1···C3	2.852 (3)	C10···H4^iv^	3.5884
O2···C1	3.571 (4)	C10···H5A^xiv^	3.3602
O2···C4	2.889 (3)	C10···H10^i^	3.4958
O2···C11	2.818 (4)	C11···H2^i^	3.4958
O4···N1	2.683 (4)	C11···H13B^x^	3.4119
O4···C14	3.576 (4)	C11···H14C^x^	3.2939
O4···C18	2.874 (3)	C11···H15A^viii^	3.1437
O5···O6	2.656 (3)	C11···H22	3.4710
O5···C20	3.425 (4)	C12···H6B^i^	3.2503
O6···O7	2.686 (3)	C12···H13B^x^	3.2962
O7···C20	3.046 (4)	C12···H14C^x^	3.3222
N1···C1	2.842 (3)	C12···H16B^v^	3.2064
N2···C14	2.889 (3)	C12···H22	3.0406
N2···C18	3.593 (4)	C13···H12A^viii^	3.4843
C1···C7	3.589 (4)	C13···H16B^v^	3.1208
C1···C8	2.768 (4)	C14···H19B^v^	3.4454
C2···C9	2.761 (4)	C15···H9^viii^	3.5099
C4···C7	2.804 (4)	C15···H19B^v^	3.0881
C4···C10	2.818 (4)	C16···H3^ix^	3.5247
C5···C9	2.773 (4)	C16···H9^viii^	3.0781
C6···C8	2.777 (4)	C16···H10^viii^	3.3785
C11···C12	3.484 (4)	C16···H12A^viii^	3.2876
C13···C16	2.769 (4)	C16···H19B^v^	3.3541
C14···C17	2.802 (4)	C17···H3^ix^	3.3226
C14···C19	2.812 (4)	C17···H12A^viii^	2.8491
C15···C18	2.800 (4)	C18···H12A^viii^	2.9718
C15···C20	3.312 (5)	C18···H16B^v^	3.0081
C17···C20	3.104 (4)	C18···H17C^v^	3.3107
C18···C21	2.824 (4)	C19···H3^i^	3.2283
O1···O2^i^	3.394 (3)	C19···H13B^vii^	3.2584
O1···C4^ii^	3.507 (4)	C20···H8^viii^	2.9211
O1···C5^ii^	3.497 (4)	C20···H9^viii^	3.4245
O1···C10^iii^	3.195 (4)	C20···H11^v^	3.2627
O2···O1^i^	3.394 (3)	C20···H13B^vii^	3.4512
O2···O8^iv^	2.829 (4)	C20···H18A^xii^	2.9457
O2···C1^i^	3.457 (4)	C20···H19B^v^	3.5820
O2···C9^i^	3.483 (4)	C21···H4^ix^	3.0689
O3···N2^i^	3.293 (3)	C21···H15A^x^	3.5799
O3···C1^ii^	3.339 (4)	C21···H17C^x^	3.3552
O3···C14^i^	3.454 (4)	C21···H21^vi^	3.5871
O4···O8	3.036 (3)	C21···H22^vi^	3.3215
O4···C20^v^	3.117 (4)	H1···O3^ii^	3.4201
O4···C21^vi^	2.995 (4)	H1···O8	2.3023
O5···O5^vii^	3.583 (3)	H1···H2^i^	3.5037
O5···C13^viii^	3.431 (4)	H1···H5A^iii^	3.1121
O5···C19^vii^	3.429 (3)	H1···H5A^ii^	3.2421
O6···N2^viii^	3.062 (3)	H1···H6B^iii^	3.3336
O6···C6^ix^	3.455 (4)	H1···H21	3.0649
O6···C11^viii^	3.485 (3)	H1···H22	2.3846
O7···C6^ix^	3.332 (4)	H2···O1^ii^	3.5296
O7···C7^ix^	3.213 (3)	H2···O8^iv^	2.6024
O8···O2^iii^	2.829 (4)	H2···C9^ii^	3.5823
O8···O4	3.036 (3)	H2···C11^i^	3.4958
O8···N1	3.111 (4)	H2···H1^i^	3.5037
O8···C1	3.214 (4)	H2···H21^iv^	2.3369
O8···C4^iii^	3.489 (4)	H2···H22^iv^	3.4017
O8···C10^ii^	3.563 (4)	H3···O6^xi^	3.2462
O8···C21^vi^	3.585 (4)	H3···O7^xi^	2.8119
N1···O8	3.111 (4)	H3···C2^ii^	3.5376
N1···C5^i^	3.564 (4)	H3···C3^ii^	3.4572
N1···C19^x^	3.408 (4)	H3···C16^xi^	3.5247
N2···O3^i^	3.293 (3)	H3···C17^xi^	3.3226
N2···O6^viii^	3.062 (3)	H3···C19^i^	3.2283
N2···C5^i^	3.435 (4)	H3···H9^i^	3.1881
N2···C19^x^	3.418 (4)	H3···H10^i^	2.8039
C1···O2^i^	3.457 (4)	H3···H12A^i^	2.4864
C1···O3^ii^	3.339 (4)	H3···H14C^i^	3.1349
C1···O8	3.214 (4)	H3···H18A^xi^	3.5468
C1···C5^ii^	3.527 (4)	H4···O6^xi^	3.5710
C2···C3^i^	3.465 (4)	H4···O7^xi^	2.5808
C2···C4^i^	3.534 (4)	H4···O8^xiii^	3.3844
C2···C8^i^	3.377 (4)	H4···C10^iii^	3.5884
C3···C2^i^	3.465 (4)	H4···C21^xi^	3.0689
C3···C3^i^	3.346 (4)	H4···H7C^iii^	2.7068
C3···C6^ii^	3.370 (4)	H4···H16B^xi^	3.3011
C3···C7^ii^	3.582 (4)	H4···H18A^xi^	3.4956
C3···C8^i^	3.520 (4)	H4···H20C^xi^	2.6541
C4···O1^ii^	3.507 (4)	H4···H21^xiii^	3.3041
C4···O8^iv^	3.489 (4)	H5A···O1^iv^	2.9971
C4···C2^i^	3.534 (4)	H5A···O1^ii^	3.1342
C4···C9^ii^	3.352 (4)	H5A···O8^iv^	3.2416
C4···C11^i^	3.228 (4)	H5A···O8^ii^	3.4717
C5···O1^ii^	3.497 (4)	H5A···C1^iv^	3.4670
C5···N1^i^	3.564 (4)	H5A···C1^ii^	3.3456
C5···N2^i^	3.435 (4)	H5A···C10^xiv^	3.3602
C5···C1^ii^	3.527 (4)	H5A···H1^iv^	3.1121
C5···C9^ii^	3.584 (4)	H5A···H1^ii^	3.2421
C5···C11^i^	3.423 (4)	H5A···H5A^xiv^	2.5047
C6···O6^xi^	3.455 (4)	H5A···H7C^xiv^	3.4116
C6···O7^xi^	3.332 (4)	H5A···H21^iv^	3.5951
C6···C3^ii^	3.370 (4)	H6B···O1^iv^	3.1272
C6···C8^ii^	3.591 (4)	H6B···O4^i^	3.4309
C7···O7^xi^	3.213 (3)	H6B···N1^i^	3.0437
C7···C3^ii^	3.582 (4)	H6B···N2^i^	3.0758
C7···C8^ii^	3.507 (4)	H6B···C12^i^	3.2503
C8···C2^i^	3.377 (4)	H6B···H1^iv^	3.3336
C8···C3^i^	3.520 (4)	H6B···H9^i^	3.4644
C8···C6^ii^	3.591 (4)	H6B···H21^i^	3.5895
C8···C7^ii^	3.507 (4)	H6B···H22^i^	3.2534
C8···C9^ii^	3.532 (4)	H7C···O1^iv^	2.9201
C8···C11^i^	3.419 (4)	H7C···O8^ii^	2.9597
C9···O2^i^	3.483 (4)	H7C···C7^iv^	3.4597
C9···C4^ii^	3.352 (4)	H7C···H4^iv^	2.7068
C9···C5^ii^	3.584 (4)	H7C···H5A^xiv^	3.4116
C9···C8^ii^	3.532 (4)	H7C···H10^i^	3.4846
C10···O1^iv^	3.195 (4)	H7C···H20C^xv^	2.9120
C10···O8^ii^	3.563 (4)	H7C···H21^ii^	3.5831
C11···O6^viii^	3.485 (3)	H7C···H22^ii^	3.2704
C11···C4^i^	3.228 (4)	H8···O6^viii^	2.6893
C11···C5^i^	3.423 (4)	H8···C4^i^	3.2662
C11···C8^i^	3.419 (4)	H8···C5^i^	3.2398
C13···O5^viii^	3.431 (4)	H8···C6^i^	3.2243
C14···O3^i^	3.454 (4)	H8···C7^i^	3.2599
C14···C15^viii^	3.417 (4)	H8···C8^i^	3.2910
C14···C16^viii^	3.557 (4)	H8···C9^i^	3.2870
C15···C14^viii^	3.417 (4)	H8···C20^viii^	2.9211
C15···C15^viii^	3.513 (4)	H8···H13B^x^	3.3359
C16···C14^viii^	3.557 (4)	H8···H15A^viii^	2.3060
C19···O5^vii^	3.429 (3)	H8···H16B^viii^	3.5914
C19···N1^xii^	3.408 (4)	H8···H17C^viii^	3.5439
C19···N2^xii^	3.418 (4)	H9···O3^i^	3.1526
C20···O4^v^	3.117 (4)	H9···O5^viii^	3.2824
C21···O4^vi^	2.995 (4)	H9···O6^viii^	2.2751
C21···O8^vi^	3.585 (4)	H9···C5^i^	3.1682
O1···H4	2.5254	H9···C6^i^	3.2112
O2···H2	2.6293	H9···C15^viii^	3.5099
O2···H8	2.5548	H9···C16^viii^	3.0781
O3···H2	2.6658	H9···C20^viii^	3.4245
O3···H3	2.4842	H9···H3^i^	3.1881
O4···H9	3.0559	H9···H6B^i^	3.4644
O4···H11	2.6258	H9···H13B^x^	2.9239
O5···H10	2.6722	H9···H14C^x^	3.5354
O5···H17C	3.0991	H9···H15A^viii^	3.4315
O7···H11	2.6708	H10···O3^i^	2.6288
O7···H16B	2.4953	H10···O6^viii^	3.4311
N1···H1	2.5246	H10···C5^i^	3.3557
N2···H8	2.3806	H10···C6^i^	3.3319
N2···H10	2.6591	H10···C10^i^	3.4958
C1···H8	3.2858	H10···C16^viii^	3.3785
C3···H1	3.2893	H10···H3^i^	2.8039
C3···H2	2.6897	H10···H7C^i^	3.4846
C3···H8	2.6729	H11···C20^v^	3.2627
C4···H3	3.2702	H11···H11^vi^	3.5697
C4···H5A	2.6739	H11···H12A^viii^	3.2547
C4···H6B	2.8310	H11···H16B^v^	2.8879
C5···H4	3.2774	H11···H17C^x^	3.0416
C5···H5A	2.5926	H11···H17C^v^	2.7454
C5···H6B	2.6464	H11···H18A^vi^	3.3895
C5···H7C	3.1970	H11···H19B^vi^	3.4714
C6···H2	3.2796	H12A···O7^viii^	3.2314
C8···H4	3.2835	H12A···C6^i^	3.4168
C9···H1	3.1871	H12A···C13^viii^	3.4843
C9···H2	3.2735	H12A···C16^viii^	3.2876
C9···H3	3.2441	H12A···C17^viii^	2.8491
C10···H2	2.5261	H12A···C18^viii^	2.9718
C11···H1	2.5886	H12A···H3^i^	2.4864
C11···H9	2.4215	H12A···H11^viii^	3.2547
C12···H10	2.6922	H12A···H18A^viii^	3.3566
C12···H11	2.6109	H13B···O5^vii^	2.5780
C13···H9	2.5219	H13B···O6^vii^	3.1493
C14···H9	2.6067	H13B···N1^xii^	3.1430
C14···H11	3.2873	H13B···N2^xii^	2.8594
C14···H12A	2.7645	H13B···C11^xii^	3.4119
C14···H14C	2.7202	H13B···C12^xii^	3.2962
C15···H12A	2.6452	H13B···C19^vii^	3.2584
C15···H13B	3.1946	H13B···C20^vii^	3.4512
C15···H14C	2.5898	H13B···H8^xii^	3.3359
C15···H17C	3.2016	H13B···H9^xii^	2.9239
C16···H10	3.2841	H13B···H13B^vii^	2.8924
C16···H11	3.2787	H13B···H15A^vii^	3.4548
C16···H15A	3.1837	H13B···H17C^vii^	3.1537
C16···H16B	2.5320	H14C···O3^i^	3.2548
C16···H17C	2.6211	H14C···O4^xii^	3.3077
C17···H16B	2.8208	H14C···N1^xii^	2.8099
C17···H17C	3.5252	H14C···N2^xii^	3.0718
C17···H18A	2.6551	H14C···C11^xii^	3.2939
C17···H19B	2.5901	H14C···C12^xii^	3.3222
C17···H20C	3.1984	H14C···H3^i^	3.1349
C18···H10	3.2877	H14C···H9^xii^	3.5354
C18···H18A	2.7626	H14C···H19B^v^	3.2867
C18···H19B	2.7518	H14C···H20C^v^	3.3909
C19···H10	2.5246	H14C···H22^xii^	3.5848
C21···H11	2.5388	H15A···O2^viii^	2.7699
C21···H16B	3.5746	H15A···O4^v^	3.0111
H1···H8	3.5095	H15A···C11^viii^	3.1437
H2···H5A	2.1969	H15A···C21^xii^	3.5799
H2···H6B	2.4330	H15A···H8^viii^	2.3060
H2···H7C	3.4914	H15A···H9^viii^	3.4315
H3···H4	2.3125	H15A···H13B^vii^	3.4548
H8···H9	2.1942	H15A···H18A^xii^	2.6808
H9···H10	2.2409	H15A···H21^v^	3.3693
H10···H12A	2.3019	H16B···O4^v^	2.7282
H10···H13B	3.4955	H16B···C12^v^	3.2064
H10···H14C	2.3167	H16B···C13^v^	3.1208
H11···H18A	2.2895	H16B···C18^v^	3.0081
H11···H19B	2.3634	H16B···H4^ix^	3.3011
H11···H20C	3.5100	H16B···H8^viii^	3.5914
O1···H2^ii^	3.5296	H16B···H11^v^	2.8879
O1···H5A^iii^	2.9971	H16B···H18A^xii^	3.3970
O1···H5A^ii^	3.1342	H16B···H19B^v^	3.5817
O1···H6B^iii^	3.1272	H17C···O4^v^	3.0931
O1···H7C^iii^	2.9201	H17C···C18^v^	3.3107
O2···H15A^viii^	2.7699	H17C···C21^xii^	3.3552
O2···H21^iv^	1.9305	H17C···H8^viii^	3.5439
O2···H22^iv^	3.1983	H17C···H11^xii^	3.0416
O3···H1^ii^	3.4201	H17C···H11^v^	2.7454
O3···H9^i^	3.1526	H17C···H13B^vii^	3.1537
O3···H10^i^	2.6288	H17C···H18A^xii^	2.3976
O3···H14C^i^	3.2548	H17C···H19B^v^	2.8980
O4···H6B^i^	3.4309	H18A···O4^vi^	2.6920
O4···H14C^x^	3.3077	H18A···C20^x^	2.9457
O4···H15A^v^	3.0111	H18A···H3^ix^	3.5468
O4···H16B^v^	2.7282	H18A···H4^ix^	3.4956
O4···H17C^v^	3.0931	H18A···H11^vi^	3.3895
O4···H18A^vi^	2.6920	H18A···H12A^viii^	3.3566
O4···H19B^vi^	3.0461	H18A···H15A^x^	2.6808
O4···H20C^vi^	2.7482	H18A···H16B^x^	3.3970
O4···H21	3.0925	H18A···H17C^x^	2.3976
O4···H22	2.2696	H18A···H21^vi^	3.4855
O5···H9^viii^	3.2824	H18A···H22^vi^	3.3830
O5···H13B^vii^	2.5780	H19B···O4^vi^	3.0461
O5···H19B^v^	3.3534	H19B···O5^v^	3.3534
O6···H3^ix^	3.2462	H19B···C14^v^	3.4454
O6···H4^ix^	3.5710	H19B···C15^v^	3.0881
O6···H8^viii^	2.6893	H19B···C16^v^	3.3541
O6···H9^viii^	2.2751	H19B···C20^v^	3.5820
O6···H10^viii^	3.4311	H19B···H11^vi^	3.4714
O6···H13B^vii^	3.1493	H19B···H14C^v^	3.2867
O7···H3^ix^	2.8119	H19B···H16B^v^	3.5817
O7···H4^ix^	2.5808	H19B···H17C^v^	2.8980
O7···H12A^viii^	3.2314	H20C···O4^vi^	2.7482
O8···H1	2.3023	H20C···O8^vi^	2.6934
O8···H2^iii^	2.6024	H20C···C7^ix^	3.5405
O8···H4^xiii^	3.3844	H20C···H4^ix^	2.6541
O8···H5A^iii^	3.2416	H20C···H7C^xvi^	2.9120
O8···H5A^ii^	3.4717	H20C···H14C^v^	3.3909
O8···H7C^ii^	2.9597	H20C···H21^vi^	2.8349
O8···H20C^vi^	2.6934	H20C···H22^vi^	2.4858
N1···H6B^i^	3.0437	H21···O2^iii^	1.9305
N1···H13B^x^	3.1430	H21···O4	3.0925
N1···H14C^x^	2.8099	H21···C3^iii^	2.9543
N1···H22	2.4483	H21···C4^iii^	3.1214
N2···H6B^i^	3.0758	H21···C8^iii^	3.4679
N2···H13B^x^	2.8594	H21···C21^vi^	3.5871
N2···H14C^x^	3.0718	H21···H1	3.0649
N2···H22	3.1769	H21···H2^iii^	2.3369
C1···H5A^iii^	3.4670	H21···H4^xiii^	3.3041
C1···H5A^ii^	3.3456	H21···H5A^iii^	3.5951
C1···H22	3.2556	H21···H6B^i^	3.5895
C2···H3^ii^	3.5376	H21···H7C^ii^	3.5831
C3···H3^ii^	3.4572	H21···H15A^v^	3.3693
C3···H21^iv^	2.9543	H21···H18A^vi^	3.4855
C4···H8^i^	3.2662	H21···H20C^vi^	2.8349
C4···H21^iv^	3.1214	H22···O2^iii^	3.1983
C5···H8^i^	3.2398	H22···O4	2.2696
C5···H9^i^	3.1682	H22···N1	2.4483
C5···H10^i^	3.3557	H22···N2	3.1769
C6···H8^i^	3.2243	H22···C1	3.2556
C6···H9^i^	3.2112	H22···C11	3.4710
C6···H10^i^	3.3319	H22···C12	3.0406
C6···H12A^i^	3.4168	H22···C21^vi^	3.3215
C7···H7C^iii^	3.4597	H22···H1	2.3846
C7···H8^i^	3.2599	H22···H2^iii^	3.4017
C7···H20C^xi^	3.5405	H22···H6B^i^	3.2534
C8···H8^i^	3.2910	H22···H7C^ii^	3.2704
C8···H21^iv^	3.4679	H22···H14C^x^	3.5848
C9···H2^ii^	3.5823	H22···H18A^vi^	3.3830
C9···H8^i^	3.2870	H22···H20C^vi^	2.4858
			
C1—O1—C9	118.55 (17)	C13—C18—C17	119.1 (3)
C5—O3—C10	116.88 (18)	H21—O8—H22	102.354
C15—O5—C19	116.87 (17)	N1—N2—H9	121.020
C16—O6—C20	113.23 (18)	C12—N2—H9	121.023
C17—O7—C21	117.20 (18)	O1—C1—H1	117.948
N2—N1—C11	115.76 (18)	C2—C1—H1	117.948
N1—N2—C12	117.96 (18)	C5—C4—H2	120.251
O1—C1—C2	124.1 (3)	C8—C4—H2	120.249
C1—C2—C3	120.6 (2)	C5—C6—H3	119.452
C1—C2—C11	120.9 (3)	C7—C6—H3	119.456
C3—C2—C11	118.44 (18)	C6—C7—H4	120.489
O2—C3—C2	122.1 (2)	C9—C7—H4	120.492
O2—C3—C8	123.1 (3)	O3—C10—H5A	109.473
C2—C3—C8	114.81 (18)	O3—C10—H6B	109.469
C5—C4—C8	119.5 (2)	O3—C10—H7C	109.473
O3—C5—C4	125.4 (2)	H5A—C10—H6B	109.469
O3—C5—C6	114.78 (19)	H5A—C10—H7C	109.473
C4—C5—C6	119.8 (3)	H6B—C10—H7C	109.469
C5—C6—C7	121.1 (2)	N1—C11—H8	119.431
C6—C7—C9	119.0 (2)	C2—C11—H8	119.431
C3—C8—C4	121.32 (19)	C13—C14—H10	120.569
C3—C8—C9	119.0 (3)	C15—C14—H10	120.565
C4—C8—C9	119.6 (2)	C13—C18—H11	120.463
O1—C9—C7	116.20 (19)	C17—C18—H11	120.485
O1—C9—C8	122.9 (2)	O5—C19—H12A	109.468
C7—C9—C8	120.9 (3)	O5—C19—H13B	109.467
N1—C11—C2	121.14 (19)	O5—C19—H14C	109.472
O4—C12—N2	123.0 (2)	H12A—C19—H13B	109.473
O4—C12—C13	122.0 (3)	H12A—C19—H14C	109.473
N2—C12—C13	114.93 (19)	H13B—C19—H14C	109.474
C12—C13—C14	121.0 (3)	O6—C20—H15A	109.471
C12—C13—C18	117.2 (2)	O6—C20—H16B	109.474
C14—C13—C18	121.8 (2)	O6—C20—H17C	109.469
C13—C14—C15	118.9 (3)	H15A—C20—H16B	109.475
O5—C15—C14	124.9 (3)	H15A—C20—H17C	109.468
O5—C15—C16	115.10 (19)	H16B—C20—H17C	109.470
C14—C15—C16	120.0 (2)	O7—C21—H18A	109.468
O6—C16—C15	119.2 (2)	O7—C21—H19B	109.474
O6—C16—C17	120.8 (3)	O7—C21—H20C	109.471
C15—C16—C17	120.1 (2)	H18A—C21—H19B	109.464
O7—C17—C16	115.1 (2)	H18A—C21—H20C	109.476
O7—C17—C18	124.8 (3)	H19B—C21—H20C	109.475
C16—C17—C18	120.1 (3)		
			
C1—O1—C9—C7	−178.53 (18)	C8—C4—C5—O3	−179.8 (2)
C1—O1—C9—C8	1.5 (4)	C8—C4—C5—C6	0.9 (4)
C9—O1—C1—C2	−1.1 (4)	H2—C4—C5—O3	0.2
C9—O1—C1—H1	178.9	H2—C4—C5—C6	−179.1
C5—O3—C10—H5A	55.8	H2—C4—C8—C3	0.3
C5—O3—C10—H6B	−64.2	H2—C4—C8—C9	179.8
C5—O3—C10—H7C	175.8	O3—C5—C6—C7	179.66 (19)
C10—O3—C5—C4	−4.8 (4)	O3—C5—C6—H3	−0.3
C10—O3—C5—C6	174.59 (19)	C4—C5—C6—C7	−0.9 (4)
C15—O5—C19—H12A	−64.3	C4—C5—C6—H3	179.0
C15—O5—C19—H13B	175.7	C5—C6—C7—C9	0.2 (4)
C15—O5—C19—H14C	55.7	C5—C6—C7—H4	−179.8
C19—O5—C15—C14	5.4 (3)	H3—C6—C7—C9	−179.8
C19—O5—C15—C16	−174.81 (17)	H3—C6—C7—H4	0.2
C16—O6—C20—H15A	173.5	C6—C7—C9—O1	−179.5 (2)
C16—O6—C20—H16B	53.5	C6—C7—C9—C8	0.5 (4)
C16—O6—C20—H17C	−66.5	H4—C7—C9—O1	0.5
C20—O6—C16—C15	104.0 (3)	H4—C7—C9—C8	−179.5
C20—O6—C16—C17	−78.1 (3)	C3—C8—C9—O1	−1.0 (4)
C17—O7—C21—H18A	65.0	C3—C8—C9—C7	179.00 (18)
C17—O7—C21—H19B	−55.0	C4—C8—C9—O1	179.5 (2)
C17—O7—C21—H20C	−175.0	C4—C8—C9—C7	−0.5 (4)
C21—O7—C17—C16	171.11 (19)	O4—C12—C13—C14	−145.3 (2)
C21—O7—C17—C18	−9.9 (4)	O4—C12—C13—C18	34.3 (3)
N2—N1—C11—C2	−176.26 (17)	N2—C12—C13—C14	35.3 (3)
N2—N1—C11—H8	3.7	N2—C12—C13—C18	−145.18 (19)
C11—N1—N2—C12	−174.65 (18)	C12—C13—C14—C15	−178.41 (18)
C11—N1—N2—H9	5.4	C12—C13—C14—H10	1.6
N1—N2—C12—O4	3.1 (4)	C12—C13—C18—C17	177.47 (17)
N1—N2—C12—C13	−177.47 (16)	C12—C13—C18—H11	−2.5
H9—N2—C12—O4	−176.9	C14—C13—C18—C17	−3.0 (4)
H9—N2—C12—C13	2.5	C14—C13—C18—H11	177.0
O1—C1—C2—C3	0.2 (4)	C18—C13—C14—C15	2.1 (4)
O1—C1—C2—C11	177.52 (19)	C18—C13—C14—H10	−177.9
H1—C1—C2—C3	−179.8	C13—C14—C15—O5	−178.63 (19)
H1—C1—C2—C11	−2.5	C13—C14—C15—C16	1.6 (3)
C1—C2—C3—O2	179.9 (2)	H10—C14—C15—O5	1.4
C1—C2—C3—C8	0.3 (3)	H10—C14—C15—C16	−178.4
C1—C2—C11—N1	16.5 (4)	O5—C15—C16—O6	−6.1 (3)
C1—C2—C11—H8	−163.5	O5—C15—C16—C17	175.94 (17)
C3—C2—C11—N1	−166.10 (19)	C14—C15—C16—O6	173.72 (19)
C3—C2—C11—H8	13.9	C14—C15—C16—C17	−4.2 (4)
C11—C2—C3—O2	2.6 (4)	O6—C16—C17—O7	4.5 (3)
C11—C2—C3—C8	−177.11 (18)	O6—C16—C17—C18	−174.60 (18)
O2—C3—C8—C4	−0.1 (4)	C15—C16—C17—O7	−177.63 (19)
O2—C3—C8—C9	−179.6 (2)	C15—C16—C17—C18	3.3 (4)
C2—C3—C8—C4	179.60 (18)	O7—C17—C18—C13	−178.71 (19)
C2—C3—C8—C9	0.1 (3)	O7—C17—C18—H11	1.3
C5—C4—C8—C3	−179.71 (19)	C16—C17—C18—C13	0.3 (4)
C5—C4—C8—C9	−0.2 (4)	C16—C17—C18—H11	−179.7

Symmetry codes: (i) −*x*+1, −*y*, −*z*+2; (ii) −*x*+2, −*y*, −*z*+2; (iii) *x*, *y*+1, *z*; (iv) *x*, *y*−1, *z*; (v) −*x*, −*y*+1, −*z*+1; (vi) −*x*+1, −*y*+1, −*z*+1; (vii) −*x*−1, −*y*, −*z*+1; (viii) −*x*, −*y*, −*z*+1; (ix) *x*−1, *y*, *z*−1; (x) *x*+1, *y*, *z*; (xi) *x*+1, *y*, *z*+1; (xii) *x*−1, *y*, *z*; (xiii) −*x*+2, −*y*+1, −*z*+2; (xiv) −*x*+2, −*y*−1, −*z*+2; (xv) *x*+1, *y*−1, *z*+1; (xvi) *x*−1, *y*+1, *z*−1.

### Hydrogen-bond geometry (Å, º)

**Table:**

*D*—H···*A*	*D*—H	H···*A*	*D*···*A*	*D*—H···*A*
O8—H21···O2^iii^	0.91	1.93	2.829 (4)	168
O8—H22···O4	0.83	2.27	3.036 (3)	153
N2—H9···O6^viii^	0.88	2.28	3.062 (3)	149
C1—H1···O8	0.95	2.30	3.214 (4)	161
C4^iii^—H2^iii^···O8	0.95	2.60	3.489 (4)	155

Symmetry codes: (iii) *x*, *y*+1, *z*; (viii) −*x*, −*y*, −*z*+1.
